# Distribution of *Anopheles gambiae* thioester-containing protein 1 alleles along malaria transmission gradients in The Gambia

**DOI:** 10.1186/s12936-023-04518-1

**Published:** 2023-03-10

**Authors:** Majidah Hamid-Adiamoh, Abdoulie Mai Janko Jabang, Kevin Ochieng Opondo, Mamadou Ousmane Ndiath, Benoit Sessinou Assogba, Alfred Amambua-Ngwa

**Affiliations:** Medical Research Council Unit The Gambia at the London School of Hygiene & Tropical Medicine, Banjul, The Gambia

**Keywords:** Thioester-containing protein (TEP)1, Distribution, *An. gambiae*, Eastern and western Gambia

## Abstract

**Background:**

Thioester-containing protein 1 (TEP1) is a highly polymorphic gene playing an important role in mosquito immunity to parasite development and associated with *Anopheles gambiae* vectorial competence. Allelic variations in *TEP1* could render mosquito either susceptible or resistant to parasite infection. Despite reports of *TEP1* genetic variations in *An. gambiae*, the correlation between *TEP1* allelic variants and transmission patterns in malaria endemic settings remains unclear.

**Methods:**

*TEP1* allelic variants were characterized by PCR from archived genomic DNA of > 1000 *An. gambiae* mosquitoes collected at 3 time points between 2009 and 2019 from eastern Gambia, where malaria transmission remains moderately high, and western regions with low transmission.

**Results:**

Eight common *TEP1* allelic variants were identified at varying frequencies in *An. gambiae* from both transmission settings. These comprised the wild type *TEP1*, homozygous susceptible genotype, *TEP1s;* homozygous resistance genotypes: *TEP1r*^*A*^ and *TEP1r*^*B*^, and the heterozygous resistance genotypes: *TEP1sr*^*A*^, *TEP1sr*^*B*^, *TEP1r*^*A*^*r*^*B*^ and *TEP1sr*^*A*^*r*^*B*^. There was no significant disproportionate distribution of the *TEP1* alleles by transmission setting and the temporal distribution of alleles was also consistent across the transmission settings. *TEP1s* was the most common in all vector species in both settings (allele frequencies: East = 21.4–68.4%. West = 23.5–67.2%). In *Anopheles arabiensis*, the frequency of wild type *TEP1* and susceptible *TEP1s* was significantly higher in low transmission setting than in high transmission setting (*TEP1*: Z = − 4.831, P < 0.0001; *TEP1s*: Z = − 2.073, P = 0.038).

**Conclusions:**

The distribution of *TEP1* allele variants does not distinctly correlate with malaria endemicity pattern in The Gambia. Further studies are needed to understand the link between genetic variations in vector population and transmission pattern in the study settings. Future studies on the implication for targeting *TEP1* gene for vector control strategy such as gene drive systems in this settings is also recommended.

## Background

*Anopheles gambiae* is the most efficient vector of *Plasmodium falciparum*, responsible for most of the malaria transmission in sub-Saharan Africa [1]. The vectorial competence of a mosquito species is dependent on its immune response to the invading parasites [2, 3], and diversity in *An. gambiae* immune-related genes modulates the variation in vector’s susceptibility to parasite transmission [4, 5]. Hence, genetic diversity of vectors could be explored in understanding its contribution to transmission dynamics of malaria across varying endemicity settings and as they adapt to interventions.

Mosquito immune response to parasite occurs in two phases: an initial response to ookinetes while crossing the midgut epithelium; and a later phase, elicited against oocysts and sporozoites in the midgut and salivary gland, respectively [6–9]. In *An. gambiae*, the initial immune response is mediated by complement-like proteins in the haemolymph, where invading *Plasmodium* is opsonized and killed [6, 8, 10]. Here, the main protein of the complement-like innate immune response is the thioester-containing protein 1 (TEP1) [10], similar to the human complement factor C3 [8, 10]. TEP1 opsonizes *Plasmodium* ookinete surface through thioester bonds and enhances lysis and melanization; inhibiting parasite development in the mosquito midgut [6, 11]. It is recognized as an important factor in genotype determining *An. gambiae* vectorial competence [6, 11].

*TEP1* gene, located on chromosome 3L, is highly polymorphic with multiple allelic variants [8, 11]. Earlier studies found variants of *TEP1* determine mosquito resistance or susceptibility to parasite. The *TEP1s* allelic variant is associated with higher susceptibility to malaria parasite infection, while *TEP1r* is linked to reduced susceptibility [7, 11, 12]. Recombinant subtypes of *TEP1s* and *TEP1r* (hybrids) which amplify the vector’s resistance to *Plasmodium* infection have also been described [8, 11].

Allelic variants of *TEP1* have been previously investigated in *An. gambiae *sensu stricto (*s.s*.) and *Anopheles coluzzii* from Mali, Burkina Faso, Ghana, Cameroon and Tanzania; where they were associated with malaria phenotypes [12]. *TEP1* variants seem to be geographically restricted and some variants are more common in a particular *Anopheles* species. *TEP1s* was most prevalent in *An. gambiae s.s.* and recorded from all countries studied. *TEP1r*^*A*^ and *TEP1r*^*B*^ which were subclusters of *TEP1r* were also identified and differentially distributed in both vector species._*.*_* TEP1r*^*B*^ was most common in *An. coluzzii* from Ghana, Mali and Burkina Faso and rarely found in the eastern African setting. *TEP1r*^*A*^ co-occurred mainly with *An. gambiae s.s.* and *An. coluzzii* at relatively low frequency. Recombinant *TEP1s/r*^*B*^ sub-type was also identified in both vectors from the three west African countries.

The distribution of *TEP1* variants is currently unknown in Gambian vector populations, where malaria transmission has significantly declined but incidence remains heterogeneous [13, 14]. During the rainy season, there are pockets of relatively high malaria transmission in the eastern region with peaks in October and November compared to the western coastal regions [13, 14]. Malaria prevalence was previously estimated to be about 19% in eastern Gambia and below 15% in the western Gambia [13, 15]. The main vectors maintaining transmission are: *An. gambiae s.s, An. coluzzii, Anopheles arabiensis* and *Anopheles melas* [16–18]*.* These vectors are widely distributed across the country, where *An. arabiensis* predominates in the east, and *An. gambiae s.s.* and *An. coluzzii* in the west of the country [16–18]. *Anopheles melas* is limited to the coastal ecosystem but extends inwards to the middle of the country during high ocean tides [18–20]. The variation in transmission intensity between the eastern and western regions in the country could also be explained by vector parity [13] and insecticide resistance [16, 21], which were reportedly higher in the eastern than the western region.

With the drive towards pre-elimination, analysis of genetic variations that could be driving local transmission may help to improve strategies for optimizing the effectiveness of interventions. Here, we determined the distribution of *TEP1* alleles in the sibling members of *An. gambiae* collected at 3 time points between 2009 and 2019 from high and low transmission settings in The Gambia.

## Methods

### Mosquito specimens

Archived genomic DNA from three previous studies: 2009 [22], 2016 [21] and 2019 [16] were analysed. These studies collected *An. gambiae* specimens during malaria transmission season (July to October) using mouth aspiration, pyrethrum spray catches and larval sampling. Specimens were collected from twenty villages that are sentinel sites of Gambian National Malaria Control Programme (GNMCP) covering the six administrative regions in The Gambia. *Anopheles gambiae* specimens were randomly selected from all sites and from each study.

### Mosquito species identification

Molecular speciation of each vector was performed by these previous studies following different previously described PCR protocols identifying *An. gambiae s.s, An. coluzzi, An. arabiensi* and *An. melas* [23, 24]. Briefly, the PCR assay involves initial amplification of ribosomal DNA specific to each of these mosquito species [23], followed by restriction enzyme digestion to discriminate *An. coluzzii*, *An. gambiae s.s*. and their hybrids (*An. coluzzii*-*An. gambiae s.s*.) [24].

### TEP1 alleles genotyping

Two *TEP1* allele-specific PCR assays were employed to genotype the variants previously described in *An. gambiae* populations [4, 12]. The first assay [4] amplifies a 428 bp fragment for the susceptible allele (*TEP1s*) and 349 bp fragment for the resistant allele (*TEP1r*). The genotypes of *TEP1r* were amplified using the second assay [12], which targets 510 bp fragment for *TEP1r*^*A*^ and 155 bp fragment for *TEP1r*^*B*^. The wild type (*TEP1*) (646 bp) was also amplified from the second assay. PCR products were analysed using QIAxcel capillary electrophoresis to identify the different fragments. Only fragments that were positive from both PCR assays were finally analysed. A total of 1400 mosquitoes were genotyped (2009 = 335; 2016 = 525; 2019 = 540).

### Data analyses

*TEP1* allele frequencies were determined as the percentage of the respective alleles in the overall vector population from each transmission setting (#allele/#total mosquito × 100). The prevalence of each vector species per setting per year was similarly calculated in percentages. The statistical differences in the proportions between the vector species and across each year were determined using Z score test. The statistical difference in the proportion of *TEP1* alleles was not compared between the transmission settings because 30% of the samples from 2009, mostly from the low setting could not be characterized. All data analyses were done using Stata/IC 16.0 (2019 StataCorp LP).

## Results

### Distribution of *An. gambiae* population along the east and west transmission settings

A total of 1,031 (74%) of the 1400 mosquitoes were successfully amplified for both species identification and *TEP1* genotyping. The DNA from these specimens could have degraded, following a decade storage. Most of the successfully amplified mosquitoes (724, 70%), were from high transmission setting (eastern Gambia) and 307 (30%) were from the low transmission setting (west). The composition of the vector species comprised *An. arabiensis*, *An. coluzzii*, *An. gambiae s.s.*, *An. melas* and hybrids of *An. coluzzii*-*gambiae s.s.* All vector species were identified from both settings except *An. melas*, which was only recorded from high transmission setting. The predominant *Anopheles* species identified in the high and the low transmission settings were *An. arabiensis* (n = 669, 65%), followed by *An. coluzzii* (n = 164, 16%), *An. gambiae s.s*. (n = 129, 13%), *An. coluzzii-gambiae s.s.* hybrids (n = 53, 5%) and *An. melas* (n = 16, 1%). *Anopheles melas* was not included in further analyses because of the low number of specimens obtained.

**Fig. 1:**
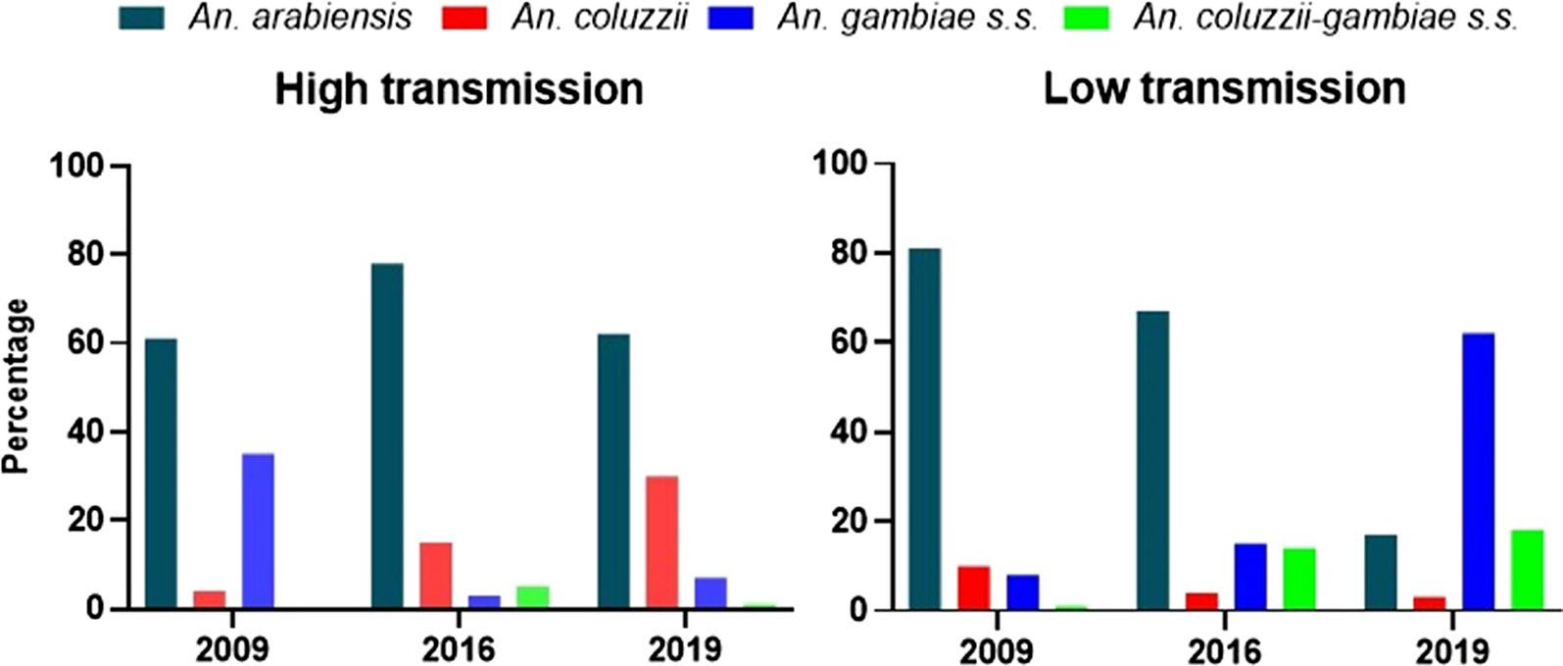
Composition of *An. gambiae* collected per year from high transmission eastern (left panel) and lower transmission western (right panel) regions of The Gambia

The composition of the local vector species was consistent in both transmission settings, where *An. arabiensis* remained predominant throughout the period except 2019 when the densities were lower in the low transmission setting (Fig. 1). There was a significant difference in the composition of the vector species per year per transmission setting (East: Z = − 4.5–15.6, P < 0.0001. West: Z = − 6.4–10, P < 0.0001).

### Distribution of *TEP1* alleles in *An. gambiae* populations across The Gambia

**Fig. 2:**
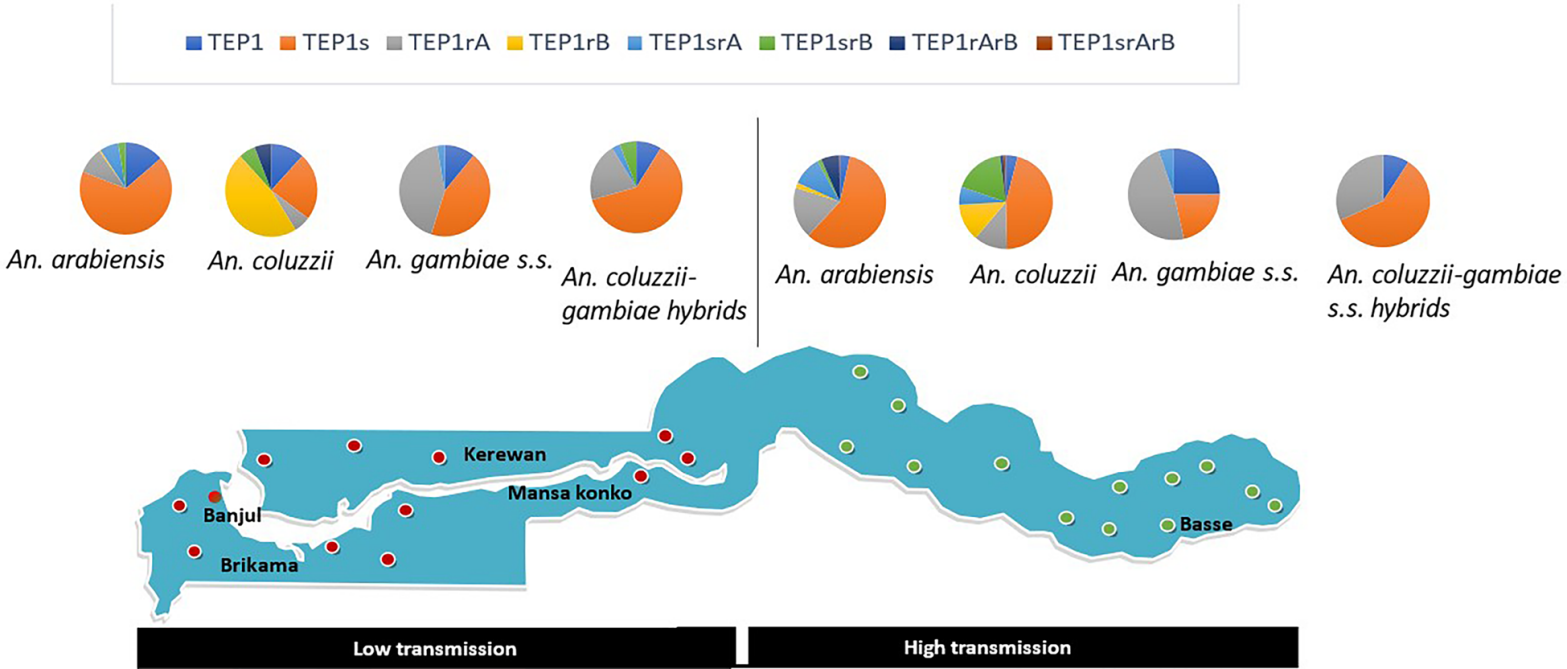
Distribution of *TEP1 alleles* in *An. gambiae* populations along the transmission gradients. TEP1 alleles from all study years were combined together in the plot. N is the total number of species Anopheles species positive for all alleles in the specific setting. The green dots inside the map indicates the study sites in the eastern Gambia, the high transmission setting while the red dots are those sites from the Western Gambia, the low transmission setting

Irrespective of the study year, eight variants of *TEP1* alleles were identified at different frequencies along the transmission settings (Fig. 2). The susceptible allele, *TEP1s* was the most prevalent in all species identified from both transmission settings. In high transmission setting, *TEP1s* was lowest in *An. gambiae s.s.* with an allele frequencies of 21.4% compared to the other vector species in the high transmission setting (Table 1). *Anopheles gambiae s.s*. from this setting mainly harboured (48.2%) the resistance *TEP1r*^*A*^ sub-type allele, while this allele was rare in other species. In the low transmission setting, *TEP1s* was the most frequent in all vector species (allele frequency: 23.5–67.2%) except *An. coluzzii* which harboured mainly (allele frequency: 47%) the *TEP1r*^*B*^ sub-type. In *An. arabiensis*, the wild type allele, *TEP1* and susceptible allele, *TEP1s* were significantly higher in the low transmission setting than the high setting (*TEP1*: Z = − 4.831, P < 0.0001. *TEPs*: Z = − 2.073, P = 0.038).

**Table 1:** TEP1 allele frequencies in vector species along the transmission gradients

		Allele frequency (%)							
		*TEP1*	*TEP1s*	*TEP1r*^*A*^	*TEP1r*^*B*^	*TEP1sr*^*A*^	*TEP1sr*^*B*^	*TEP1r*^*A*^*r*^*B*^	*TEP1sr*^*A*^*r*^*B*^
High transmission	*An. arabiensis* (N = 486)	3.5	58	17.9	3.3	10	1.4	6.4	0.2
	*An. coluzzii* (N = 147)	4	45.6	11.6	13	6	17.7	1.4	0.7
	*An. gambiae s.s.* (N = 56)	25	21.4	48.2	0	5.4	0	0	0
	*An. coluzzii-gambiae s.s.* (N = 19)	10.5	68.4	21	0	0	0	0	0
Low transmission	*An. arabiensis* (N = 183)	13.7	67.2	9.3	0.5	6.6	2.7	0	0
	*An. coluzzii* (N = 17)	11.8	23.5	5.9	47	0	5.9	5.9	0
	*An. gambiae s.s.* (N = 73)	11	43.8	42.5	0	2.7	0	0	0
	*An. coluzzii-gambiae s.s.* (N = 34)	8.8	61.8	20.6	0	2.9	5.9	0	0

Allele frequency was calculated as a percentage of the allele in overall vector populations irrespective of collection year. N is the total number of all *TEP1* alleles detected in the specific Anopheles species

Heterozygous *TEP1* genotypes, *TEP1sr*^*B*^, *TEP1r*^*A*^*r*^*B*^ and *TEP1sr*^*A*^*r*^*B*^ were highly prevalent in *An. arabiensis* and *An. coluzzii* but rare in *An. gambiae s.s.* from both settings (Fig. 2). Only *TEP1sr*^*A*^ was found in *An. gambiae s.s.* (genotype frequency: East = 5.4%, West = 2.7%).

### Dynamics of *TEP1* diversity in vector species along the transmission gradients

**Fig. 3:**
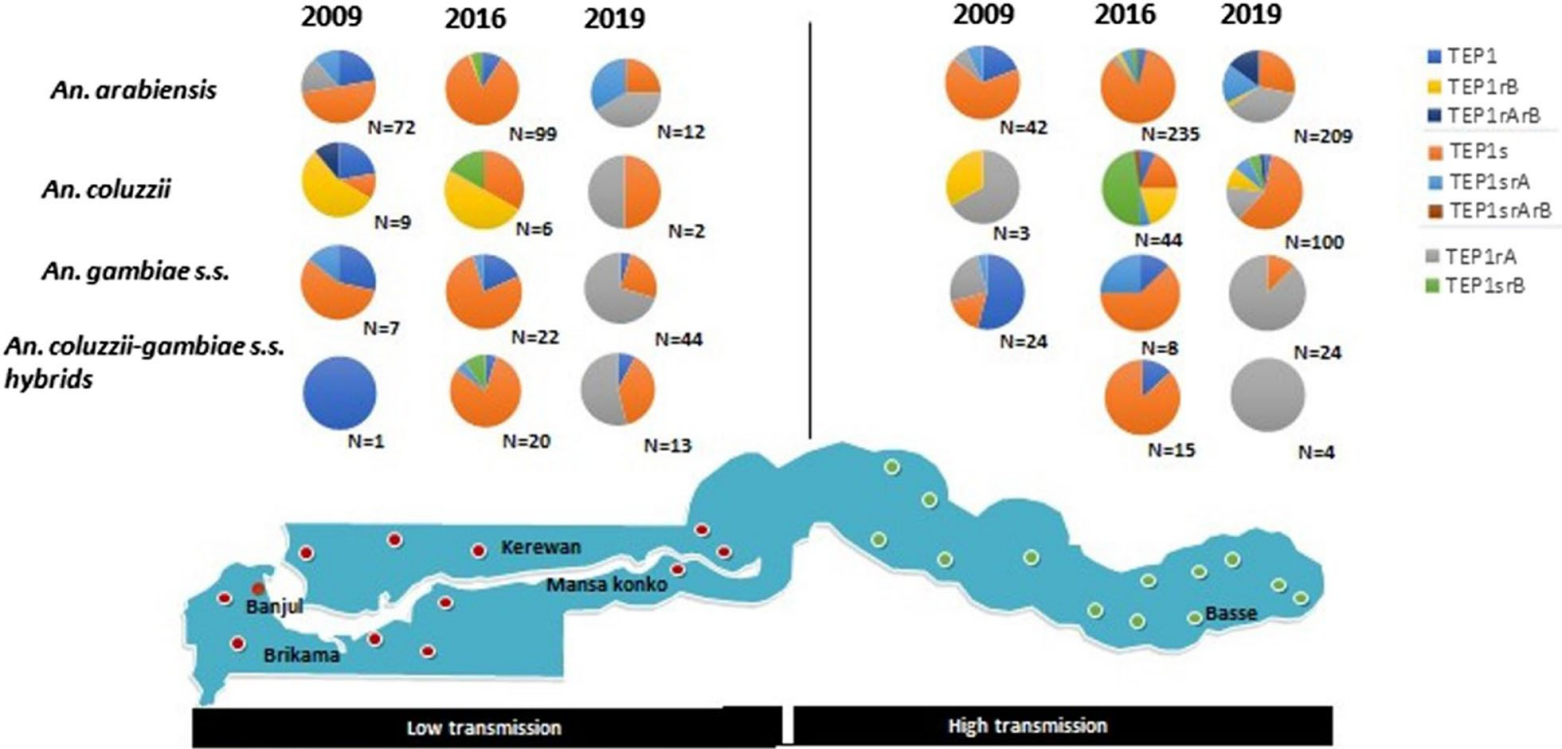
Dynamics of *TEP1 alleles* in *An. gambiae* populations over time along the transmission gradients. TEP1 alleles from all study years were separated by year. The green dots inside the map indicates the study sites in the eastern Gambia, the high transmission setting while the red dots are those sites from the Western Gambia, the low transmission setting

The pattern of allele diversity was temporally consistent in each vector species identified. The majority of the *TEP1* alleles detected in 2009 were still present in 2016 and 2019 at both settings (Table 2 and Fig. 3). Most of the species harboured *TEP1s* which was consistently the most frequent allele over time at both settings; and the frequency steadily increased in *An. coluzzii* over the years especially in high transmission setting (allele frequencies: 2009 = 0%; 2016 = 18.2%; 2019 = 59%). *TEP1r*^*A*^ was uncommon in all vector species prior to 2019 in both settings. *TEP1r*^*B*^ was equally uncommon and was detected in *An. arabiensis* and *An. coluzzii in* the high transmission setting in 2019 (allele frequencies 1.9% and 9%, respectively).

**Table 2:** Dynamics of TEP1 diversity in vector species along the transmission gradients

	High transmission (Allele frequency, %)								Low transmission (Allele frequency, %)							
	TEP1	TEP1s	TEP1rA	TEP1rB	TEP1srA	TEP1srB	TEP1rArB	TEP1srArB	TEP1	TEP1s	TEP1rA	TEP1rB	TEP1srA	TEP1srB	TEP1rArB	TEP1srArB
2009																
*An. arabiensis* (N:E = 42, W = 72)	19	67	7	0	7	0	0	0	22.2	50	16.7	0	11	0	0	0
*An. coluzzii* (N: E = 3, W = 9)	0	0	66.7	33.3	0	0	0	0	22.2	11.1	0	55.6	0	0	11.1	0
*An. gambiae s.s* (N: E = 24, W = 7)	54	17	25	0	4	0	0	0	28.6	57	0	0	14.3	0	0	0
*An. col-gam s.s* (N: E = 0, W = 1)	0	0	0	0	0	0	0	0	100	0	0	0	0	0	0	0
2016																
*An. arabiensis* (N:E = 235, W = 99)	3.8	83.8	2.6	2.1	4.3	3	0	0.4	9	85	0	1	0	5	0	0
*An. coluzzii* (N: E = 44, W = 6)	6.8	18.2	0	20.5	4.5	47.7	0	2.3	0	33.3	0	50	0	16.7	0	0
*An. gambiae s.s*. (N: E = 8, W = 22)	12.5	62.5	0	0	25	0	0	0	18.2	77.3	0	0	4.5	0	0	0
*An. col-gam s.s*. (N: E = 15, W = 20)	13.3	86.7	0	0	0	0	0	0	5	80	0	0	5	10	0	0
2019																
*An. arabiensis* (N: E = 209, W = 12)	0	28.2	37.3	1.9	17.7	0	14.8	0	0	25	41.7	0	33.3	0	0	0
*An. coluzzii* (N: E = 100, W = 2)	3	59	15	9	7	5	2	0	0	50	50	0	0	0	0	0
*An. gambiae s.s*. (N: E = 24, W = 44)	0	12.5	87.5	0	0	0	0	0	4.5	25	70.5	0	0	0	0	0
*An. col-gam s.s*. (N: E = 4, W = 13)	0	0	100	0	0	0	0	0	7.7	38.5	53.8	0	0	0	0	0

Heterozygous *TEP1* genotypes: *TEP1sr*^*B*^, *TEP1r*^*A*^*r*^*B*^ and *TEP1sr*^*A*^*r*^*B*^ were not detected in the vectors from both settings in 2009. *TEP1r*^*A*^*r*^*B*^ was identified mainly in *An. arabiensis* and *An. coluzzii* in 2019 in high transmission setting.

## Discussion

As malaria transmission is relatively higher in eastern than western Gambia [13, 14], determining if genetic variation in the local vector populations could be partly driving this difference could improve our understanding on local vector competence. This knowledge is highly important as The Gambia aims for pre-elimination phase. This study characterized *TEP1* gene alleles in vector populations collected at 3 time points within a decade from different regions of The Gambia. Eight previously described variants were found at varying frequencies across the country. There was no distinct restriction of specific *TEP1* alleles by vector species or transmission setting; as the distribution of *TEP1* alleles was similar and consistent in the vectors from both transmission settings across the study periods. However, the alleles were recorded at heterogenous levels, with the susceptible allele (*TEP1s*) predominating in both settings across years*.*

Lack of clustering of *TEP1* alleles to specific vector species or transmission setting could be explained by similar composition of vector species in both settings and selective pressure on this gene that is maintained overtime. More importantly, these findings may imply that *TEP1* plays limited role in the heterogeneous prevalence of malaria in The Gambia. Previous studies [17, 22, 29] have attributed the variance in transmission to insecticide resistance, which is currently higher in vectors in high transmission setting. Additionally, other studies [16–18] suggested that the relatively higher abundance of highly efficient vectors in the high transmission setting could be a factor. Further studies are needed to understand the role of other possible factors including human and vector behaviour [30, 31], as well as environment factors [32, 33]. The implication of targeting *TEP1* gene for vector control in this setting also requires further investigations.

The pattern of distribution of *TEP1* recorded between high and low transmission setting is consistent with a recent study in western Kenya [25], which did not document any significant difference in *TEP1* alleles in vector populations in study areas comprising high and low transmission settings. The study suggested that the expanded vector control interventions in the study areas could have impacted the genetic structure of the vector population as documented in other settings [26, 27]. Vector interventions were scaled up during the period studied here, with a shift from DDT to pirimiphos methyl for IRS; as well as high coverage of LLINs throughout the country [28]. This indicates that despite this selection pressure against the vectors, there was no significant temporal shifts in alleles on *TEP1* locus, and the effect of insecticides on the overall genetic structure of the natural vector population is unclear.

The observed high frequency of the resistant allele (*TEP1r*^*A*^) along with low frequency of the susceptible allele (*TEP1s*) in the highly competent vector, *An. gambiae s.s*. in high transmission setting prompts future investigations to further understand the role of adaptive interaction between this vector and parasite populations in this setting. Earlier studies have shown that adaption of the circulating *P. falciparum* populations to *TEP1*-mediated killing in sympatric *An. gambiae* population may be a driving factor for endemicity of malaria in sub-Saharan Africa [34]. Already, *TEP1r*^*A*^ and *TEP1s* were previously associated with low and high infection prevalence respectively in specific parasite-vector populations [4, 34], suggesting ongoing local host-parasite adaption, which can be investigated in future with concomitant generation of parasite prevalence and genetic data.

This study recorded similar patterns of *TEP1r*^*A*^ and *TEP1r*^*B*^ in *An. gambiae s.s.* and *An. coluzzii,* respectively, where *TEP1r*^*A*^ was common in both vectors but *TEP1r*^*B*^ was exclusively found in *An. coluzzii*, as previously documented from Mali, Burkina Faso, Ghana, Cameroon and Tanzania [12]. However, the frequencies of these resistance-associated alleles were generally low and similar in both transmission settings. Taken together *TEP1* alleles are not specifically affected by dwindling parasite biomass or interventions against the vectors.

Allelic recombination was common in *An. arabiensis* and *An. coluzzii* as previously shown [4, 11, 12], and consistently higher overtime in the high transmission setting than the low transmission setting. High recombination events in *An. gambiae TEP1* gene was suggested to promote higher infection prevalence relative to homozygote *TEP1r* allele [11]. The relatively higher *TEP1* heterozygous genotypes observed in *An. arabiensis* and *An. coluzzii* in the higher transmission setting could possibly be a factor for better vector competence in both vectors. However, lack of enough studies linking genetic diversity to vector competence in this setting hampers the interpretation of this finding. Nevertheless, sporozoite rates were recently reported higher in these vectors than *An. gambiae s.s.* in this setting [16]. A future study investigating the transmission efficiency of the vector species with the identified *TEP1* alleles would better explain these results.

The temporal distribution of *TEP1* alleles was consistent across the transmission settings in all vector populations. However, the consistent increase in *TEP1s* in *An. coluzzii* in both settings also prompts further phenotypic interrogations designed to determine the effect of these alleles in vector competence and malaria prevalence.

This study was limited by the inability to determine sporozoite infection rates in the vector population due to lack of adequate samples. Also, low number of specimens from some years and regions hampered the power to statistically compare the distribution of variants and malaria rates across the country and by specific year. However, the observed vector composition and densities were consistent with the previous studies, where vectors are relatively more abundant in the high than low transmission setting [16, 17, 21].

## Conclusions

This study presented baseline data on *TEP1* allelic variants of *An. gambiae* in The Gambia. Eight variants were identified at consistently similar pattern in both transmission settings and overtime. The susceptible allele was common in most vector species and transmission setting. These findings open opportunities for further studies to understand genetic changes in vectors populations that could be driving the current transmission pattern in The Gambia and implication for consideration of *TEP1* for malaria control strategy such as gene drive systems in this setting.

## Data Availability

All relevant data are within the paper. No supporting Information is available.

## Abbreviations

TEP1: Thioester-containing Protein 1
DNA: Deoxyribonucleic acid
IRS: Indoor residual spraying
LLIN: Long-lasting insecticidal net
PCR: Polymerase chain reaction
